# Improved Object Detection Using a Robotic Sensing Antenna with Vibration Damping Control

**DOI:** 10.3390/s17040852

**Published:** 2017-04-13

**Authors:** Vicente Feliu-Batlle, Daniel Feliu-Talegon, Claudia Fernanda Castillo-Berrio

**Affiliations:** 1 Escuela Técnica Superior de Ingenieros Industriales, Universidad de Castilla-La Mancha, 13071 Ciudad Real, Spain; 2 Instituto de Investigaciones Energéticas y Aplicaciones Industriales (INEI), Campus Universitario de Ciudad Real, 13071 Ciudad Real, Spain; Daniel.Feliu@uclm.es; 3 Engineering and Architecture Faculty, Mechatronic Engineering, Istanbul Gelisim University, Cihangir mah. Sehit Jandarma Komando Er Hakan Oner Sk. No. 1 Avcilar, 34351 Istanbul, Turkey; claudiafer54@gmail.com

**Keywords:** robotic sensor, obstacle recognition, sensing antenna, flexible robot, motion control, active vibration damping, impact detection

## Abstract

Some insects or mammals use antennae or whiskers to detect by the sense of touch obstacles or recognize objects in environments in which other senses like vision cannot work. Artificial flexible antennae can be used in robotics to mimic this sense of touch in these recognition tasks. We have designed and built a two-degree of freedom (2DOF) flexible antenna sensor device to perform robot navigation tasks. This device is composed of a flexible beam, two servomotors that drive the beam and a load cell sensor that detects the contact of the beam with an object. It is found that the efficiency of such a device strongly depends on the speed and accuracy achieved by the antenna positioning system. These issues are severely impaired by the vibrations that appear in the antenna during its movement. However, these antennae are usually moved without taking care of these undesired vibrations. This article proposes a new closed-loop control schema that cancels vibrations and improves the free movements of the antenna. Moreover, algorithms to estimate the 3D beam position and the instant and point of contact with an object are proposed. Experiments are reported that illustrate the efficiency of these proposed algorithms and the improvements achieved in object detection tasks using a control system that cancels beam vibrations.

## 1. Introduction

In recent years, there has been an increasing interest in developing integrated sensory systems, especially relating to the various tactile sensors [1]. Multiple applications entail integrated systems, as is the case of machine assemblies for precise positioning, impact protection, navigation, etc. Tactile/touch sensing is essential in developing human-machine interfacing and electronic skins for areas such as automation, security and medical care [2].

Tactile sensors were first explored in the early 1990s, for example, in the works of Russell [3]. Natural tactile sensors, such as whiskers and antennae, have been explored in recent works, e.g., [4]. Several attempts have been made to build bio-mimetic active sensory applications, which are also known as vibrational systems. Multiple forms of engineering applications based on mammal or insect-inspired sensing have been researched, such as the work in [5], in which whisker-based texture discrimination on a mobile robot is presented. The less frequent use of tactile sensing may be partly attributed to its complex and distributed nature. Some problems, such as placement, robustness of sensors or wiring complexity, among others, make its effective utilization difficult.

In the last two decades, a robust and compact sensor device has been proposed that efficiently solves some of these problems, which is called a sensing antenna. It is an active sensor that consists of a flexible-beam moved by some servo-controlled motors and a load-cell placed between the beam and the motors. This device replicates the touch sensors that some animals possess and performs an active sensing strategy in which the servomotor system moves the beam back and forth until it hits an object. At this instant, information of the motor angles combined with force and torque measurements allow us to calculate the positions of the hit points, which are valuable information about the object surface. A 3D map of the surface of an object, which allows its recognition, can be obtained using this device. Two strategies can be applied to obtain such 3D maps. The first one is to keep moving the beam back and forth in order to hit the object at different points, which allows one to determine their 3D coordinates, and then extract the map of the object surface. This strategy is carried out by some insects that use their antennae for this purpose (e.g., [6]). The other one is to slide the beam across the object, exerting a controlled force on the surface of the object in order not to lose contact, and collect the 3D coordinates of points on the object surface during this movement. This strategy is utilized by some mammals that have whiskers as sensors (e.g., [7]). These two strategies can be implemented using the aforementioned sensing antennae.

The idea of using artificial antenna-based sensors as tactile sensors has been previously studied in, e.g., [8,9]. In [10], the contact information was acquired by processing the vibration signals produced in the mechanical structure of the antenna during its movement in a recognition task, since the vibration frequencies of the antenna change from the free motion case to the constrained motion case and also as a function of the location of the contact point. In contrast, in [9], the static deflection of the antenna was used in combination with a nonlinear elastic equation to obtain the contact point.

However, multiple constrains limit the design of these devices and the performance of their movements, such as the flexible-beam length, light weight and flexibility. These characteristics make the dynamic behavior of these antennae quite complicated, making it difficult to achieve fast and precise movements. If the control system in charge of moving the motors does not consider these dynamics, i.e., the beam elasticity, residual vibrations appear that prevent the accurate and fast approach to the target points to be searched. Moreover, permanent collisions with the object could happen, where the antenna hits back and forth. These phenomena would produce delays in the recognition process, diminish the quality of the estimates of the object surface and reduce therefore the efficiency of the device functioning.

The dynamics of the flexible-beam implies a non-minimum phase behavior if a non-collocated input-output pair is considered (e.g., the dynamics existing between the tip position and the torques provided by the motors), whose model is described by high order coupled non-linear differential equations. In addition, the system is driven by small servo-motors in which the limited torques, intermittent operation and the strong non-linearity owing to the static friction constrains the motor control design. In this system, the stability of the closed-loop control is additionally sensitive to non-modeled dynamics and parameter uncertainties.

Active control approaches have shown that closed-loop control of the antenna can be used to sample a three-dimensional space, direct the antenna tip towards some targets and search for special points in a precise manner so as to provide the maximum amount of task-relevant information. This would imply additional control efforts, such as high tip-position accuracy and residual vibration damping for precise tasks. Beyond this, the ability to work with active sensing approaches in different situations may constitute a major achievement. Few works on active tactile sensing have considered the system constraints, such as the servo-motor drivers, beam elasticity, controllers, computer system, data processing, etc., in the design of the positioning control system.

Several studies suggest that the performance of an antenna for sensing tasks significantly depends on its accurate and fast position and/or force control [7,11]. This could save time during the object recognition process and allow specific inspection areas of an object. The way the antenna approaches an object is very important, along with the precision of the sensor measurement when the contact is established. In spite of its importance, this issue has not been dealt with in depth in previous bio-inspired tactile sensors.

The accurate detection of the instant at which the collision of the antenna with the object is produced is of utmost importance in order to reduce the time needed to estimate the 3D coordinates of the contacted point. In the context of robotics, several mechanisms have been proposed that detect a collision when the absolute value of some measured variables exceeded a threshold: [12] for rigid link robots and [13,14,15] for flexible link robots. In this article, we propose to exploit the feature that our antenna is a single link mechanism that has a very low mechanical inertia in order to develop simplified algorithms for the detection of the contact instant.

The combination of the above collision detectors with force controllers allows one to perform complex object recognition tasks, like the ones carried out by whiskers that slide on surfaces. These tasks require the change of the control system, when the contact happens, from a tip position control to a hybrid control in which some directions are position controlled and others force controlled. Some research has been carried out about such control systems in the context of flexible link robots. In [13], a force control scheme for a single link flexible robot was proposed, which was activated by an impact detection mechanism. Control laws that switch between them as a function of a collision detection mechanism were proposed in [14] for a single link flexible arm. A hybrid position-force control of a sensing antenna that slides on a surface recognizing an object by carrying out a repetitive control was proposed in [16]. This strategy is effective, but is limited by the cyclical nature of the movements to be performed. Moreover, it did not implement any collision detection mechanism and, then, the transition from free to constrained movements could not be efficiently done.

In order to study the aforementioned issues and improve the positioning performance of sensing antennae, we have designed, built and tested several experimental prototypes. We have tested antennae constituted by different motor configurations and flexible beams. In [17], a prototype of flexible link antenna was designed, built and tested. The configuration of the motors was chosen in such a way that the volume swept by the actuators configuration was minimum in order to allow an easier installation of this device on-board a mobile robot. It was shown that the antenna with such a motor configuration behaved as a two link rigid-flexible robot. Moreover, a small mass was added at the tip of the antenna in order to obtain a single significant vibration mode and simplify then the vibration control of the link. The dynamic model proposed for this antenna was verified experimentally, and a new motor control for this system was also developed and tested. However, a closed-loop control of the antenna vibrations was not developed in this article. In [18], another antenna prototype was developed that had a less compact motor configuration than the previous one, but it had a simpler dynamic model that facilitated the closed-loop control, since it behaved as a single flexible link robot. A small mass was also placed at the tip of the link, which was 0.55 m long, in order to simplify the link dynamics, as was done in the previous prototype. A new closed-loop control system was developed to simultaneously control the antenna tip position and remove the link vibrations, which was based on a double nested control loop (the inner loop was the motor control loop developed in the previous article) and a feedback linearization technique. Experiments were reported that proved the satisfactory performance of the system. In [19], a simpler control system was tested in this antenna. It combined a feedforward term that inverted the antenna dynamics with the previous two nested control loops, in which PD controllers were used for the outer loop. However, although placing a mass at the tip simplifies the antenna dynamics, it also slows down the movements of the antenna. Since antennae with much longer links are better suited for mobile robot navigation with obstacle detection, a link 0.98 m long without a mass at its tip was used in order to achieve faster motions with larger links in [20]. In this article, a motor control loop was closed (the inner loop), while the outer loop used in the previous systems was substituted by a nonlinear input shaper in order to drive the link in an open-loop manner. This prototype is the one used in the present article. Finally, in [21], a dynamic model of our large link with distributed mass and without a payload at its tip was proposed, which included three vibration modes. Moreover, a two nested closed-loop control system was proposed in which the inner loop was the already utilized motor control. The outer loop, which was in charge of damping the vibrations of the three modes, combined a feedforward term with a robust fractional-order controller, in order to avoid spillover effects. All of these articles were devoted to control the free movements of the two prototypes of antennae and cancel the vibrations that appeared in the link during the motion. Contact tasks were not studied in any of these papers.

The previous platforms include a load-cell sensor, which is a force-torque (F-T) sensor that provides information and sensory feedback to the controllers and allows active touch sensing. Measurements of this sensor were used in the previous papers only to close the loop that canceled the vibrations. The present article uses the measurements of this sensor in active touch tasks. The antenna configuration and the control system developed in [21] constitute the starting point of this research. This paper proposes new mechanisms to: (1) estimate in real time the 3D position of any point of the antenna moving in the free space; (2) estimate the instant at which the impact of the antenna with an object is produced; and (3) estimate the point of the antenna at which the contact has been produced, by using the (F-T) sensor and the motor encoders’ measurements. Moreover, this article aims to demonstrate that canceling the vibrations with a closed-loop control system in the case that the antenna moves freely improves the accuracy of the estimates in active touch tasks.

In this paper, Section 2 describes the laboratory setup. The dynamic model of the antenna prototype and its control system are outlined in Section 3. The new estimation algorithms are developed in Section 4. Results obtained with these algorithms are described in Section 5. A discussion of these results is presented in Section 6, and some concluding remarks are given in Section 7.

## 2. Materials

### 2.1. Mechanism Characteristics

**Figure 1:**
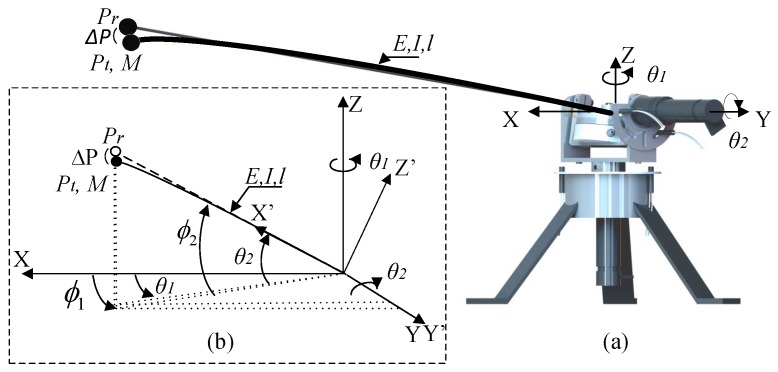
2DOF flexible-beam sensor: (**a**) mechanism design and (**b**) schematic diagram.

The motor configuration is shown in Figure 1a, which illustrates the mechanism that is used to hold the multi-axis (F-T) sensor. Two servo-motor sets (motor, gear-box and encoder) are used to drive the sensor, and there is a flexible-beam on the top of the sensor whose initial point at the base of the beam coincides with both motor shafts. Throughout this paper, the subscript “*i*” denotes the motors, variables, mechanical parameters and controllers associated with a particular degree of freedom. The subscript i=1 refers to the azimuthal angle degree of freedom and i=2 to the elevation angle degree of freedom.

Figure 1b shows the schematic diagram in which the equivalent length of the beam is *l*. The beam deflection was limited to 10% of the total beam length in order to obtain a linear beam deflection. Larger or non-linear deflection is not studied in this work. $P_{t}$ is the tip of the flexible-beam, and $P_{r}$ is the beam tip itself when the beam is considered to be a rigid-beam. Δ*P* is a 3D vector that describes the beam deflection; *E* is the Young modulus; *I* is the inertial moment resulting from the flexible-beam cross-section; *g* is the gravity constant (9.81 $\frac{m}{s^{2}}$); and *M* is the tip mass. The tip position is expressed in spherical coordinates $\phi_{1}$ and $\phi_{2}$ with regard to the absolute Cartesian frame (*X*,*Y*,*Z*). The rigid part of the system, which rotates the motor angles $\theta_{1}$, $\theta_{2}$, holds the (F-T) sensor and the flexible-beam that is attached to one of its two sides. This allows the Cartesian coupling force ${\vec{F}}_{s}=$ $\left(F_{x},F_{y},F_{z}\right)$ and torque ${\vec{\Gamma }}_{s}=$($\Gamma_{x}$,$\Gamma_{y}$,$\Gamma_{z}$) be measured. In this work, the tip position is estimated using the measured torques at the base of the beam and the motor angles. The tip position is expressed in the frame (X,Y,Z), and the frame that eventually results from the rigid mechanism rotations is ($X^{\prime },Y^{\prime },Z^{\prime }$). Moreover, coupling forces and torques can be represented in this last frame as ${\vec{F}}_{s^{\prime }}$ and ${\vec{\Gamma }}_{s^{\prime }}$, respectively.

**Figure 2:**
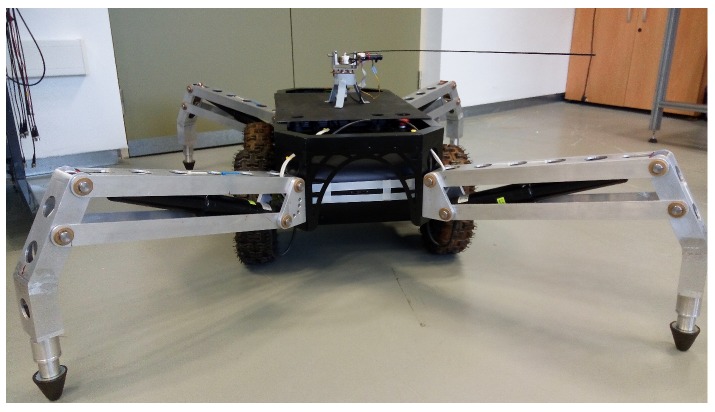
Antenna prototype with a flexible beam, mounted on a legged-wheeled mobile robot.

Figure 2 shows the antenna device studied in this article. It has a very lightweight beam without any payload attached at its end. Since this device is lighter than other previous prototypes, it can be moved faster, which signifies that it has the capability of collecting more points on the surface of the object in a given time. However, the dynamic model of this beam is relatively complex, which involves that sophisticated control systems are required to perform accurate movements.

### 2.2. Hardware

**Figure 3:**
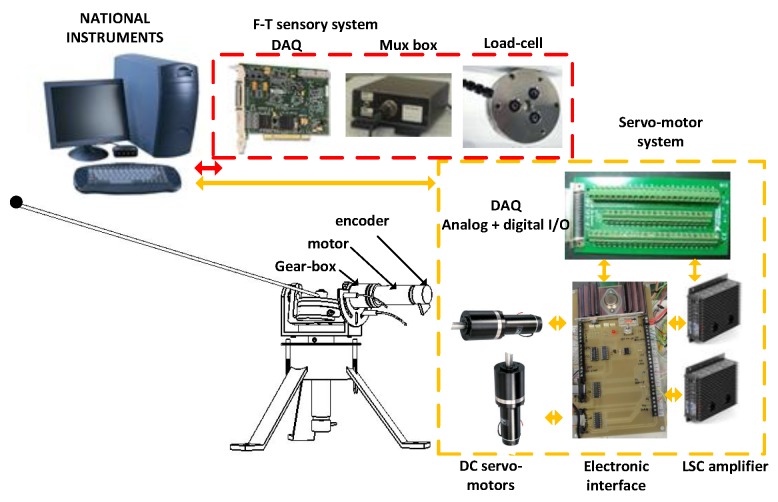
Experimental platform: system setup.

Figure 3 shows the system setup, which includes the mechanism, the servo-motor system, the (F-T) sensory system, electronic interfaces and a basic computer to run the system. In order to put together and install each of the necessary component parts, a basic card for an adequate connection was designed (electronic interface), which includes: (1) the servo-motor connectors; (2) servo amplifier connectors; (3) input/output connections of the DAQ; (4) inputs of the limit switches; (5) digital inputs implemented with optocouplers that allow the system to stop automatically when the opening limit-switches are reached; and (6) two voltage sources necessary to account for the variation in power consumption ±5 V and ±18 V.

### 2.3. Software Requirements

The system runs under Microsoft Windows 7, 2009, Intel (R) Core (TM) i7 CPU, 2.8 GHz 8 GB of RAM. The controllers were implemented using a basic computer with a limited sample time $T_{s}=1$ ms. The dynamics of both beams (with and without mass at its tip) were slow in comparison to this sample time, signifying that it is possible to use this digital computer implementation in a straightforward manner. This system includes a digital card NI PCI-6229 for data acquisition (DAQ), which is used for the analog control signals and encoder counters of two linear servo-motors, along with digital inputs from four limit switches.

Model simulations were carried out using MATLAB/Simulink R2010a and were compared to the results of several experiments in order to validate the parameter identification and the system modeling. The motor parameter identification was accomplished by using the MATLAB Parameter Estimation Toolbox. Moreover, a finite element software was used to run simulations of movements of the antennae and to verify the accuracy of the proposed dynamical models.

### 2.4. Servo-Motor System

The experimental platform is driven by two PMA-5A DC mini servo-motor sets. Each set includes a zero backlash reduction gear (ratio *n* = 100) and an incremental encoder. The reduction rate “*n*” increases the torque of the system while diminishing its velocity. It also reduces the effect of the motor-beam coupling torque, thus making it negligible at the motor shaft. Each servo-motor is powered by a current linear servo controller (LSC) 30-2 that controls the input current to the servo-motor. This input current is proportional to the control signal supplied by the computer. The LSC is a 4-quadrant (4-Q) servo amplifier powered by permanent magnets. This servo amplifier is used to control DC motors up to approximately 50 W and allows modes such as the following to be controlled: (1) I × R compensation; (2) voltage regulator; (3) digital encoder control; (4) DC tacho-control; and (5) current control. The current control loop keeps the motor current (torque) at a predetermined set value and is suitable for applications with an external position controller. A wide input DC voltage of 12–30 V makes the LSC very flexible as regards being used with different voltage sources and is well suited to fixed speed adjustment. The required mode is the current control, which is easily selected by using a dip switch. The current regulator was adjusted by obtaining a zero offset and limiting the maximum current, which should be below the maximum permissible continuous current of the servo-motors (motor data sheet).

### 2.5. Sensory System

The servo-motor angles, $\theta_{1}$ and $\theta_{2}$, were measured using encoders whose precision is $7\times 10^{-5}$ rad. The system makes use of an ATI Mini 40 (F-T) sensor, transducer hardware and a digital card NI PCI-6220 for data acquisition. The (F-T) sensor was used to measure the coupling torque, $\Gamma_{1}^{coup}$ and $\Gamma_{2}^{coup}$, as well as the the coupling force, $F_{1}^{coup}$ and $F_{2}^{coup}$, between the motors and the beam, at the base of the beam. These measurements are used to: (1) implement a beam position control system; (2) estimate the beam position during free movements; and (3) estimate the instant of the contact and the point of the beam at which it is produced.

A camera-based optotrack system was also used as an external sensor. This required three infrared cameras, which measure the 3D antenna tip position in real time. The optotrack precision is 0.1 mm, which is useful to verify that our control scheme removes the first two vibration modes of the link of the antenna. It is remarked that this measure is only used to check the antenna tip behavior. It is not used in the closed-loop control.

### 2.6. Flexible-Beam Parameters

The beam is very flexible and is made of carbon fiber. Its length was determined according to the 2DOF work space dimension desired by the foreseen application. The basic beam parameters are listed in Table 1. This table also includes the frequency of the first vibration mode and the damping ratio, which represent the resistance to the transversal tip velocity associated with this mode. Most of these parameters will be used for the antenna modeling in the next Section 3.1 and were measured or calculated on the basis of the antenna material and geometry. Curve fitting methods to experimental data were performed using the MATLAB/Simulink Parameter Estimation toolbox to determine the best fitting parameters, such as the Young modulus and the damping ratio.

## 3. Methods

### 3.1. Antenna Dynamics

#### 3.1.1. Dynamics of the DC Motors

The dynamics of the DC motors is represented in a compact matrix form using a differential equation of second order:

(1) $$\hat{\underline{\Gamma }}=\hat{\underline{K}}\cdot \underline{\vartheta }=\hat{\underline{J}}\cdot \ddot{\hat{\underline{\theta }}}+\hat{\underline{\upsilon }}\cdot \dot{\hat{\underline{\theta }}}+{\hat{\underline{\Gamma }}}^{nlf}+{\hat{\underline{\Gamma }}}^{coup}$$

where the motor control signals: $\underline{\vartheta }={\left(\vartheta_{1},\vartheta_{2}\right)}^{T}$ are the system inputs, $\underline{\theta }={\left(\theta_{1},\theta_{2}\right)}^{T}$ are the motor outputs, $\hat{\underline{K}}=diag({\hat{K}}_{1},{\hat{K}}_{2})$ are the motor constants, $\hat{\underline{J}}=diag({\hat{J}}_{1},{\hat{J}}_{2})$ are the rotor motor inertias and: $\hat{\underline{\upsilon }}=diag\left({\hat{\upsilon }}_{1},{\hat{\upsilon }}_{2}\right)$ are the viscous friction coefficients. The symbol “·” denotes differentiation with regard to time and “*T*” matrix transpose. The motors are endowed with fast dynamics linear servo amplifiers, signifying that the current of the motors and the motor torques: $\hat{\underline{\Gamma }}={\left({\hat{\Gamma }}_{1},{\hat{\Gamma }}_{2}\right)}^{T}$ are proportional to the previous control signals in which $\hat{\underline{K}}$ defines the gains. The motor torques caused by non-linear static friction are denoted by: ${\hat{\underline{\Gamma }}}^{nlf}={\left({\hat{\Gamma }}_{1}^{nlf},{\hat{\Gamma }}_{2}^{nlf}\right)}^{T}$. The term ${\hat{\underline{\Gamma }}}^{coup}={\left({\hat{\Gamma }}_{1}^{coup},{\hat{\Gamma }}_{2}^{coup}\right)}^{T}$ represents the coupling torque between the beam and motor shafts. In our antennae, the coupling torque can be considered negligible owing to the use of reduction gears $\underline{n}=diag\left(n_{1},n_{2}\right)$ with high ratios.

Magnitudes seen from the motor side of the gear are written with an upper hat. Magnitudes seen from the beam side are denoted using standard letters. Angles seen from the motor side are transformed using the gear ratio $n_{i}$: ${\hat{\theta }}_{i}=n_{i}\theta_{i}$, while the torque conversion is given by ${\hat{\Gamma }}_{i}=\frac{\Gamma_{i}}{n_{i}}$.

Table 2 shows the parameters of the motors, where $\vartheta_{s,i}$ is the saturation voltage of the motors and $\vartheta_{i}^{nlc}={\hat{\Gamma }}_{i}^{nlf}/{\hat{K}}_{i}$ the Coulomb friction of the motors in terms of voltage. Note that $\vartheta_{i}^{nlc}$ is 40% of the saturation limit $\vartheta_{s,i}$. Then, Coulomb friction is very noticeable in our motors.

#### 3.1.2. Beam Dynamics

The dynamics of a flexible beam with distributed mass has infinite vibration modes, which yield an infinite order model. Usually, it is truncated, yielding an approximate reduced order model.

**Figure 4:**
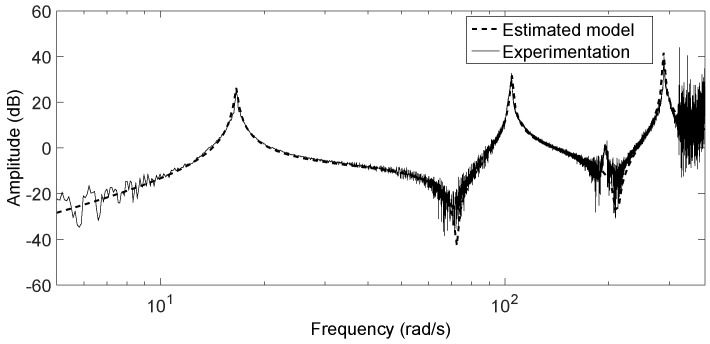
Identification of the model.

In order to get a model as precise as possible, an identification process has been carried out on the experimental antenna. This can be performed in different ways. Among these, the method described in [22] was employed here because it is easy to use and is well suited for systems with little damped and very decoupled vibration modes. A chirp signal was applied to the input, which stimulated the different modes of vibration in azimuthal movements. This signal has a range of frequencies from 0.2 Hz–80 Hz in 80 s. Carrying out this process on the experimental antenna, we noticed that only the three first modes of vibration were excited. The relationship between the coupling torque $\Gamma_{1}^{coup}$ and the motor angle $\theta_{1}$ was characterized. The magnitude of the frequency response obtained in experimentation between these two variables is shown in Figure 4. A transfer function $G_{\Gamma }\left(s\right)=\Gamma_{1}^{coup}\left(s\right)/\theta_{1}\left(s\right)$ was fitted to these data:

(2) $$G_{\Gamma }\left(s\right)=\frac{0.37s^{2}}{s^{2}+0.3s+16.4^{2}}+\frac{0.372s^{2}}{s^{2}+0.84s+104^{2}}+\frac{0.75s^{2}}{s^{2}+4.6s+289^{2}}$$

whose zeros are 0 (double), $z_{1},z_{1}^{*}=-0.27\pm j72.2$ and $z_{2},z_{2}^{*}=-1.3\pm j211.4$, and whose poles are −0.15±j16.4, −0.4±j104 and −2.3±j289. The magnitude of the frequency response of Equation (2) is also drawn in Figure 4. This figure shows that the experimental dynamics are accurately approximated by model Equation (2) with three vibration modes. Furthermore, it is observed that the sensor measurements are very noisy from 50 Hz–80 Hz.

Detailed analysis of this beam and experimentation have shown that in the antenna movements:

- The vibration associated with the first mode is much more relevant than the vibrations associated with the other modes.
- In the usual movements of the antenna, the Coriolis and centripetal torques are much smaller than the inertial torques, and they can therefore be neglected (see [20]).
- The previous item allows us to assume that the azimuthal and attitude dynamics are approximately decoupled in our antenna.
- However, the gravitational torque is significant in the attitude component of the movements.
- Link deflection is small compared to the link length (less than 10%), allowing us to assume a linear model of the deflection.

Consequently, the beam dynamics is modeled as if the azimuthal and attitude movements were decoupled and were described by the same transfer function Equation (2). The attitude model is completed by adding the effect of gravity on the associated coupling torque, which is $-\frac{3EIg}{l^{2}\omega_{1}^{2}}\cos\left(\phi_{2}\right)$ (this term was obtained in [21]).

### 3.2. Control System

In this subsection, the control system of our antenna device is described. Measurements of the motor positions $\underline{\theta }$ and the torque at the base of the link ${\underline{\Gamma }}^{coup}$ are fed back. The control strategy consists of two nested control loops (named the inner and outer loops) for each movement. The inner loop combines a motor position controller with a linear feedback of the measurements of the (F-T) sensor, the coupling torque ${\underline{\Gamma }}^{coup}$, to make the dynamics between the motor angle and its reference, denoted as ${\underline{\theta }}^{*}$, robust to the Coulomb and viscous frictions of the motors and to link parameter changes, thus allowing one to consider it as a linear time-invariant system. The outer loop feeds back the coupling torque $\Gamma_{i}^{coup}$, and it is in charge of canceling the link vibration.

#### 3.2.1. Inner Loop

**Figure 5:**
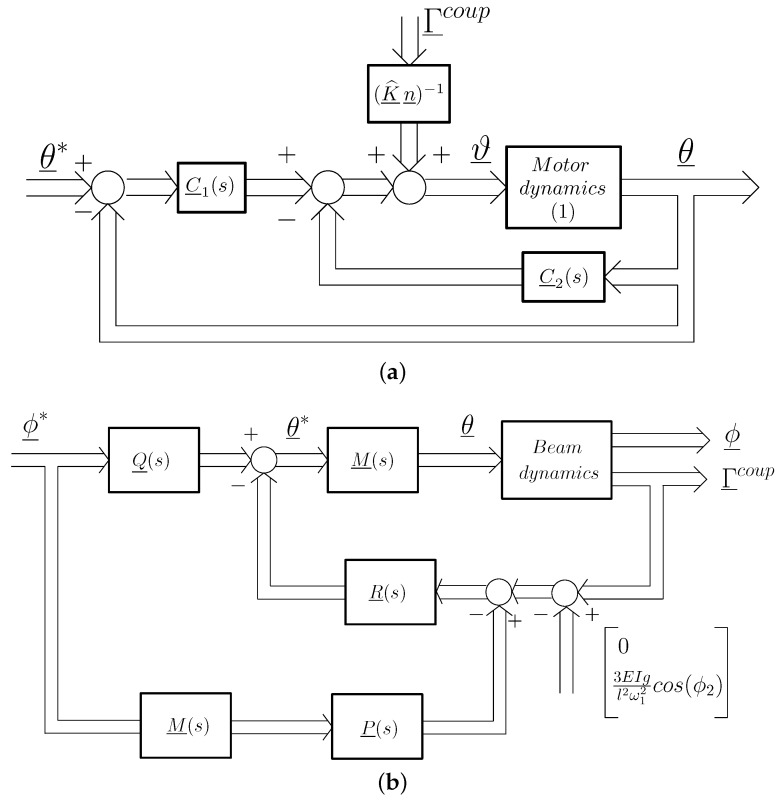
(**a**) Inner loop; (**b**) outer loop.

PID controllers with a low pass filter term have been used because they ensure good trajectory tracking, compensate disturbances such as unmodeled components of the friction and are robust to parameter uncertainties, providing precise and fast motor positioning responses. The proposed control law is:

(3) $$\underline{\vartheta }\left(s\right)={\underline{C}}_{1}\left(s\right)\cdot \left({\underline{\theta }}^{*}\left(s\right)-\underline{\theta }\left(s\right)\right)-{\underline{C}}_{2}\left(s\right)\cdot \underline{\theta }\left(s\right)+{\left(\underline{\hat{K}}\cdot \underline{n}\right)}^{-1}\cdot {\underline{\Gamma }}^{coup}\left(s\right)$$

where it is noted that the last term cancels the influence of the beam on the motor dynamics. A block diagram of this control law is shown in Figure 5a. The controllers are of the form ${\underline{C}}_{1}\left(s\right)=diag\left(C_{1,1}\left(s\right),C_{1,2}\left(s\right)\right)$ and ${\underline{C}}_{2}\left(s\right)=diag\left(C_{2,1}\left(s\right),C_{2,2}\left(s\right)\right)$ being:

(4) $$C_{1,i}\left(s\right)=\frac{a_{2,i}s^{2}+a_{1,i}s+a_{0,i}}{s(s+h_{i})};\;C_{2,i}\left(s\right)=\frac{b_{1,i}s+b_{0,i}}{s+h_{i}},\;i=1,2$$

The parameters of these controllers can be easily tuned by using an algebraic method in order to place the four closed-loop poles and the two zeros of each loop in desired locations, while maintaining zero steady-state error to step disturbances (Coulomb friction can be considered to some extent a disturbance of this type) and step commands. The four poles of each closed-loop are placed in the same position: $p_{i}$. Moreover, the poles of the two loops are made equal: $p_{1}=p_{2}=-60$. The two zeros of each loop are placed also in −60 in order to cancel two of the poles and then obtain closed-loop transfer functions with constant numerators. The algebraic method that allows designing such controllers was developed in [21] and yields the controller parameter values shown in Table 3.

Then, the closed-loop transfer functions that result for the two motors are the same:

(5) $$M_{i}\left(s\right)=\frac{\theta_{i}\left(s\right)}{\theta_{i}^{*}\left(s\right)}=\frac{1}{{\left(1+\kappa_{i}\cdot s\right)}^{2}}\;;\;\;\kappa_{i}={\left(-p_{i}\right)}^{-1}=1/60,\;\;i=1,2.$$

#### 3.2.2. Outer Loop

The outer loop control system is devoted to canceling all of the vibrations of the antenna. It is sketched in Figure 5b, which shows that the feedback loop is closed with the coupling torque ${\underline{\Gamma }}^{coup}$ measured by the (F-T) sensor. In this figure:

- The trajectory desired for the tip position is ${\underline{\phi }}^{*}={\left({\phi_{1}}^{*},{\phi_{2}}^{*}\right)}^{T}$.
- The motor control loop (inner loop) is represented by $\underline{M}\left(s\right)=diag\left(M_{1}\left(s\right),M_{2}\left(s\right)\right)$.
- The feedback controller is $\underline{R}\left(s\right)=diag\left(R_{1}\left(s\right),R_{2}\left(s\right)\right)$.
- A feedforward term is $\underline{Q}\left(s\right)=diag\left(1+\frac{s^{2}}{\omega_{1}^{2}},1+\frac{s^{2}}{\omega_{1}^{2}}\right)$, where $\omega_{1}=16.4$ rad/s is the frequency of the first vibration mode, which eliminates this frequency component of the references in order to not excite this first vibration mode in the beam.
- A signal $\underline{P}\left(s\right)\underline{M}\left(s\right){\underline{\phi }}^{*}\left(s\right)$, being $\underline{P}\left(s\right)=\frac{3EI}{l}\left(\underline{Q}\left(s\right)-\underline{I}\right)$ and $\underline{I}$ the 2 × 2 identity matrix, represents the torque component required to perform the above feedforward control action, which is subtracted from the coupling torque (see [21]).
- The lower part of the figure shows that a quantity is subtracted from the torque associated with the attitude degree of freedom in order to remove the component of the torque caused by gravity and use only the torque component caused by the beam vibrations in the feedback.

Controllers $R_{1}\left(s\right)$ and $R_{2}\left(s\right)$ should remove the residual vibrations of the beam. Their task is to damp the three vibration modes shown in Figure 4 and the Transfer Function (2). PI controllers are often used in flexible robot control because they are robust to spillover effects (e.g., [23]). However, though the closed-loop system does not become unstable with these controllers, the second and third modes are barely damped. In order to keep spillover robustness and effectively damp the three vibration modes that are apparent in the beam spectrum, a fractional order phase lag compensator was proposed in [21]. The methodology to design this controller was developed in that paper, and it is subsequently outlined.

The proposed fractional-order phase-lag controller for the outer loop is of the form:

(6) $$C\left(s\right)={\left(K_{c}\frac{1+T\cdot s}{1+T\cdot f\cdot s}\right)}^{\alpha }\;,$$

where the exponent α is a positive real number α>0, $K_{c}>0$, T≥0 and f≥1. This controller generalizes the standard phase-lag compensators often employed to control flexible structures (these are the particular case α=1 of Equation (6)). It was demonstrated in [21] that the compensator that minimizes the sensor high frequency noise on the outer loop control signal is given by:

(7) $$C\left(s\right)=\rho {\left(\frac{\sqrt{1+\eta^{2}}}{1+\frac{\eta }{\omega_{c}}\cdot s}\right)}^{\alpha }\;,$$

where η and ρ are the parameters of the controller and $\omega_{c}$ is the desired gain crossover frequency.

**Figure 6:**
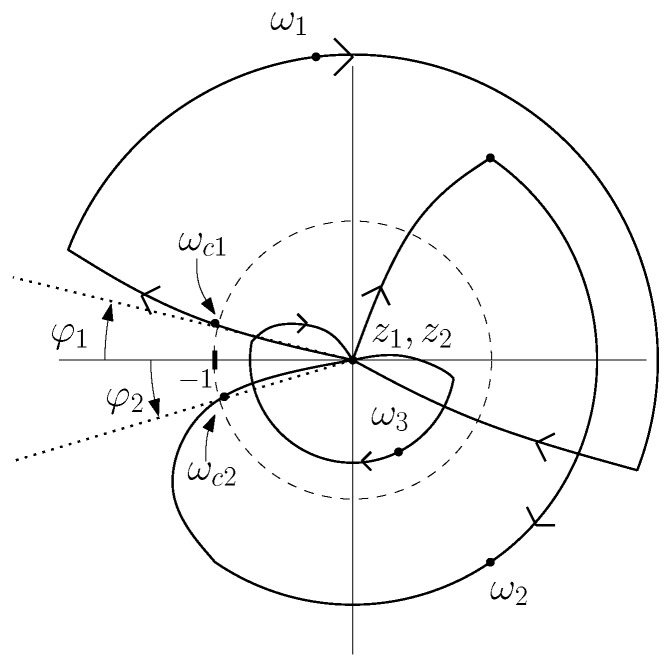
First path of the Nyquist diagram (0≤ω<∞).

The open-loop transfer function of the system is described now by $L\left(s\right)=G_{\Gamma }\left(s\right)M\left(s\right)C\left(s\right)$. The design method is based on a frequency response technique. Then, it is important to bear in mind the first path of the Nyquist diagram (0<ω<∞) of L(s), which is depicted in Figure 6. It can be observed in this diagram that there are at least two phase margins $\varphi_{1}$, $\varphi_{2}$, with their corresponding gain crossover frequencies $\omega_{c1}$ and $\omega_{c2}$, which are associated with the first and second vibration modes, respectively. According to Model (2), there could be a third phase margin, associated with the third vibration mode, but our controller is designed in such a way that the magnitude of L(jω) is less than unity for frequency values $\omega >|z_{2}|$, the spillover effects therefore being prevented.

Due to the fact that the fractional-order controllers have two design conditions ($\varphi_{1}$, $\omega_{c1}$) and three parameters to be tuned, the process to calculate the parameters of the controllers is as follows. Given a value of α and conditions ($\varphi_{1}$, $\omega_{c1}$), calculate the two remaining parameters of the controller (7). Controllers with the form of Equation (7) were pursued with fractional orders comprised in the interval α∈[0,2], which fulfil the following three objectives: (a) robustness to spillover effects, i.e., the second and third vibration modes must not unstabilize the closed-loop system; (b) the second vibration mode must be damped; and (c) a settling time of the dynamics associated with the first vibration mode lower than a desired value must be guaranteed in order to get fast tracking of the tip trajectory and fast disturbance rejection. Then, a region of feasible fractional-order controllers was defined, which verified simultaneously several specifications in the frequency and time domains while guaranteeing the absence of spillover, in order to fulfill the three above objectives.

It was demonstrated in [21] that an integer-order controller of the form (7) (with α=1) does not exist that fulfills all of the previous specifications. In fact, it was stated that the condition of guaranteeing a minimum value of $\varphi_{2}$ in order to obtain a significant damping in the second mode forces the controller to have an order α below 1. A fractional-order controller structure provides therefore more flexibility to the design methodology than the integer-order one in order to attain the above proposed control objectives.

Finally, a controller belonging to the above-defined region of feasible controllers was chosen by an optimization procedure that maximized a combined cost of the relative dampings of the first and second vibration modes. This methodology was shown to be effective to design the controllers of our antenna and can also be applied to other systems having several vibration modes, like flexible robots, flexible structures or micro/nanopositioning systems. The controllers yielded by this procedure for our antenna are:

(8) $$R_{1}\left(s\right)=R_{2}\left(s\right)=\frac{2.16}{{\left(1+0.14\cdot s\right)}^{0.55}}$$

**Figure 7:**
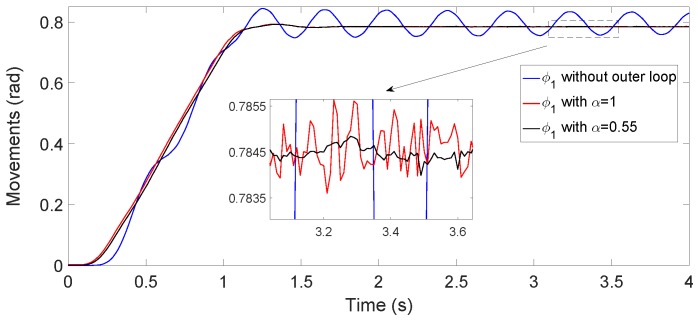
Controlled azimuthal movement.

Experiments demonstrated that the residual vibration in the positioning of the antenna tip using the fractional-order controller is ten-times lower than using the equivalent integer-order controller (in the sense of both controllers yielding the same phase margin and gain crossover frequency associated with the first vibration mode) because the fractional-order controller is able to damp the second vibration mode, whereas the integer-order controller is not. This fact was demonstrated carrying out several azimuthal, attitude and combined azimuthal-attitude antenna movements. Figure 7 compares the performances of these equivalent integer-order and fractional-order phase-lag controllers and shows the higher damping of the second mode achieved by the second controller. These responses were experimentally obtained by using the optotrackexternal sensor mentioned in Section 2.5.

The method to implement such a fractional-order controller in a computer was also detailed in [21].

## 4. Estimation Mechanisms

This subsection develops new mechanisms to detect the contact of the antenna with an object.

### 4.1. Estimation of the Antenna Position during Free Movement

The antenna has to be moved in a controlled way to reach the regions that have to be explored. Then, it is necessary to know in real time the position in the space of all the points of the beam in order to prevent undesired collisions. Moreover, the control algorithm that implements the outer loop uses estimates of the tip position, e.g., the torque value that is subtracted in the measurement in Figure 5b in the attitude degree of freedom needs the estimate of $\phi_{2}$. This subsection develops a simple, but efficient estimator of the beam position using motor encoders and (F-T) sensor measurements.

Consider a flexible beam with distributed mass, which is clamped at one of its ends, and the other end vibrates freely in a horizontal plane (without gravity effects). The deflection of the beam at each position *x* and time *t* can be expressed (e.g., [24]) as $w\left(t,x\right)=\sum_{k=1}^{\infty }\psi_{k}\left(x\right)\cdot \delta_{k}\left(t\right)$. In this expression, each term $\psi_{k}\left(x\right)\cdot \delta_{k}\left(t\right)$ represents the *k*-th vibration mode. In the case of a clamped-free ends beam, we have that:

(9) $$\psi_{k}\left(x\right)=\frac{\sin\left(\beta_{k}l\right)-\sinh\left(\beta_{k}l\right)}{\cos\left(\beta_{k}l\right)+\cosh\left(\beta_{k}l\right)}\left(\sin\left(\beta_{k}x\right)-\sinh\left(\beta_{k}x\right)\right)+\cos\left(\beta_{k}x\right)-\cosh\left(\beta_{k}x\right),\;0\leq x\leq l,$$

where the $\beta_{k}$ are the multiple solutions of the equation $\cos\left(\beta_{k}\cdot l\right)\cdot \cosh\left(\beta_{k}\cdot l\right)=-1$. Moreover, we have that $\delta_{k}\left(t\right)=A_{k}\sin\left(\omega_{k}t+\phi \right)$, being $\omega_{k}=\beta_{k}^{2}\sqrt{(E\cdot I)/\rho }$.

It is also verified that:

(10) $$\Gamma^{coup}\left(t\right)=-EI\sum_{k=1}^{\infty }\psi_{k}^{\prime \prime }\left(0\right)\cdot \delta_{k}\left(t\right),\;\psi_{k}^{\prime \prime }\left(0\right)={\left.\frac{\partial^{2}\psi_{k}\left(x\right)}{\partial x^{2}}\right|}_{x=0}$$

We make the assumption that only the first vibration mode is relevant in an azimuthal movement. Taking into account that $w_{i}\left(t,l\right)=l\left(\phi_{i}\left(t\right)-\theta_{i}\left(t\right)\right)$ (the subscript “*i*” in w(t,x) denotes deflection in a particular degree of freedom), it is obtained that:

(11) $$w_{1}\left(t,l\right)=-\frac{\psi_{1}\left(l\right)}{\psi_{1}^{\prime \prime }\left(0\right)}\frac{\Gamma_{1}^{coup}\left(t\right)}{EI}\Rightarrow \phi_{1}\left(t\right)=\theta_{1}\left(t\right)-\frac{\psi_{1}\left(l\right)}{EIl\psi_{1}^{\prime \prime }\left(0\right)}\Gamma_{1}^{coup}\left(t\right)$$

which allows one to estimate the tip azimuthal angle $\phi_{1}$ from the angle of the first motor $\theta_{1}$ and the component of the coupling torque $\Gamma_{1}^{coup}$ associated with the azimuthal movement. In the case that the antenna is not horizontal, but it has an attitude angle $\theta_{2}$, the previous expression is modified to:

(12) $$w_{1}\left(t,l\right)=-\frac{\psi_{1}\left(l\right)}{\psi_{1}^{\prime \prime }\left(0\right)}\frac{\Gamma_{1}^{coup}\left(t\right)}{EI\cos(\theta_{2}\left(t\right))}\Rightarrow \phi_{1}\left(t\right)=\theta_{1}\left(t\right)-\frac{\psi_{1}\left(l\right)}{EIl\psi_{1}^{\prime \prime }\left(0\right)\cos\left(\theta_{2}\left(t\right)\right)}\Gamma_{1}^{coup}\left(t\right)$$

The deflection in an attitude movement is more complex because gravity is acting in a distributed way along the beam. Then, we approximate the tip position in an attitude movement by the first vibration mode, as in the azimuthal movement, adding a disturbance term caused by gravity:

(13) $$w_{2}\left(t,l\right)=-\frac{\psi_{1}\left(l\right)}{\psi_{1}^{\prime \prime }\left(0\right)}\frac{\Gamma_{2}^{coup}\left(t\right)}{EI}+\mu l\cos\left(\theta_{2}\left(t\right)\right)\Rightarrow \phi_{2}\left(t\right)=\theta_{2}\left(t\right)-\frac{\psi_{1}\left(l\right)}{EIl\psi_{1}^{\prime \prime }\left(0\right)}\Gamma_{2}^{coup}\left(t\right)+\mu \cos\left(\theta_{2}\left(t\right)\right)$$

where μ is determined by fitting this model to the simulations carried out with a finite elements software package. Estimators (11) and (13) can be easily extended to estimate the deflection of any point of the beam at a distance *x* of its clamped end:

(14) $$w_{1}\left(t,x\right)=-\frac{\psi_{1}\left(x\right)}{\psi_{1}^{\prime \prime }\left(0\right)}\frac{\Gamma_{1}^{coup}\left(t\right)}{EI\cos(\theta_{2}\left(t\right))};\;w_{2}\left(t,x\right)=-\frac{\psi_{1}\left(x\right)}{\psi_{1}^{\prime \prime }\left(0\right)}\frac{\Gamma_{2}^{coup}\left(t\right)}{EI}+\mu x\cos\left(\theta_{2}\left(t\right)\right)$$

### 4.2. Estimation of the Instant of Contact

Estimating the instant of contact is of utmost importance because: (1) when a contact is produced, either the trajectory or/and the control mode (position or force control) has to be switched in order to prevent damages in the antenna or the object; and (2) the mechanism in charge of detecting the contacted point of the beam, which will be described in the subsequent subsection, is triggered. We propose in this subsection a new mechanism to detect the contact instant.

Consider the coupling torque ${\vec{\Gamma }}_{s^{\prime }}\left(t\right)$ provided by the (F-T) sensor in the Cartesian rotated frame ($X^{\prime }$,$Y^{\prime }$,$Z^{\prime }$) shown in Figure 1b. Denote the effect of gravity on the beam, measured by the (F-T) sensor as a force ${\vec{F}}_{s^{\prime }}^{g}=$ $\left(-mg\sin\left(\theta_{2}\right),0,-mg\cos\left(\theta_{2}\right)\right)$ and a torque ${\vec{\Gamma }}_{s^{\prime }}^{g}=$ $\left(0,0.5mgl\cos(\theta_{2}),0\right)$, where *m* is the mass of the link (see Table 1). Denote as ${\vec{\Psi }}_{s^{\prime }}\left(t\right)$ the real-time estimation of the coupling torque yielded by an observer of the first vibration mode, which is obtained by implementing the first term of Equation (2):

(15) $$\Psi_{i}\left(s\right)=\frac{0.37s^{2}}{s^{2}+0.3s+16.4^{2}}\theta_{i}\left(s\right),\;i=1,2$$

where $\Psi_{1}\left(t\right)$ gives the torque component in the $Z^{\prime }$ axis, $-\Psi_{2}\left(t\right)$ the component in the $Y^{\prime }$ axis and the torque component in the $X^{\prime }$ is zero because there is no torsion in the beam. This observer can be easily implemented since its inputs are the measured motor angles $\theta_{1}\left(t\right)$ and $\theta_{2}\left(t\right)$. The approximate observation of the coupling torque is completed by adding to ${\vec{\Psi }}_{s^{\prime }}\left(t\right)$ the torque produced by the gravity ${\vec{\Gamma }}_{s^{\prime }}^{g}$. Denote the residual error between the measured and the estimated coupling torques as:

(16) $$r\left(t\right)={\vec{\Gamma }}_{s^{\prime }}\left(t\right)-\left({\vec{\Psi }}_{s^{\prime }}\left(t\right)+{\vec{\Gamma }}_{s^{\prime }}^{g}\left(t\right)\right)$$

Then, a contact is produced at the instant at which the absolute value of the time derivative of the magnitude of the filtered residual error vector exceeds a threshold:

(17) $$\left|\frac{dr_{m}^{f}\left(t\right)}{dt}\right|>\varepsilon$$

where $r^{f}\left(t\right)$ is the result of passing the components of the vector r(t), separately, through a linear filter $F\left(s\right)=1/{\left(1+0.004s\right)}^{2}$, and $r_{m}^{f}\left(t\right)$ is the magnitude of the vector $r^{f}\left(t\right)$.

### 4.3. Estimation of the Point of Contact

Estimating the point of contact of the beam gives the 3D information about the point on the surface of the object that has been hit, which is needed to perform the object detection/recognition task. Once the contact has been produced, velocities and accelerations of the antenna are very small. Then, the movement of the antenna is quasi-static, and the forces and torques measured by the (F-T) sensor are the reaction of the object and the effect of the gravity on the antenna. In this case, the contact point $x_{c}$ on the beam can be approximated by the algorithm:

(18) $$x_{c}\approx \frac{\left|{\vec{\Gamma }}_{s}-{\vec{\Gamma }}_{g}\right|}{\left|{\vec{F}}_{s}-{\vec{F}}_{g}\right|}$$

This algorithm is valid only if no friction force is produced during the contact.

## 5. Results

### 5.1. Simulated Results on Beam Position Estimation

The beam position estimators proposed in Equation (14) are tested in azimuthal and attitude trajectories in which the tip moves $45^{\circ }$ in 1.2 s. In these trajectories, only motor control is used (only the inner loop is closed), in order to assess how the estimator approximates the beam oscillations. Once the coefficients of Equation (14) have been calculated from the mechanical properties of the beam, estimators of the tip deflection are obtained:

(19) $$w_{1}^{e}\left(t,l\right)=-\frac{3.0253}{\cos(\theta_{2}\left(t\right))}\Gamma_{1}^{coup}\left(t\right),\;w_{2}^{e}\left(t,l\right)=-3.0253\Gamma_{2}^{coup}\left(t\right)+0.0098\cos\left(\theta_{2}\left(t\right)\right)$$

An estimator proposed in [18] is also tested for comparison purposes, which is based on the assumption that the beam is massless, i.e., all of the beam mass is lumped at its tip. In this case, the beam deflection in an azimuthal movement is given [25] by $w_{1}\left(t,l\right)=-\frac{l^{2}}{3EI}\Gamma_{1}^{coup}\left(t\right)$. Once its coefficients have been calculated, the following estimators of the tip deflection are obtained:

(20) $$w_{1}^{e}\left(t,l\right)=-\frac{2.632}{\cos(\theta_{2}\left(t\right))}\Gamma_{1}^{coup}\left(t\right),\;w_{2}^{e}\left(t,l\right)=-2.632\Gamma_{2}^{coup}\left(t\right)$$

Moreover, a finite element program has been used as a testbed, in which our beam was modeled by 100 flexible elements.

**Figure 8:**
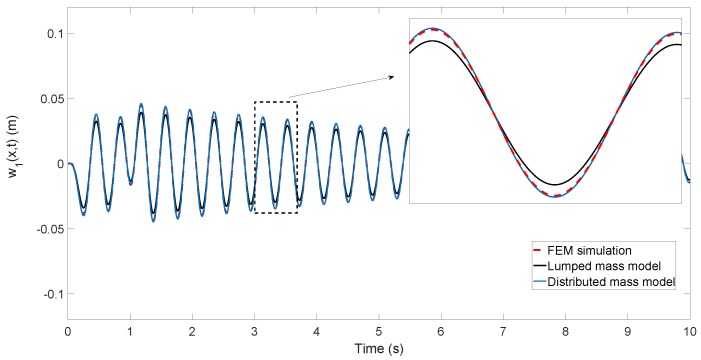
Estimation of the tip deflection in an azimuthal movement (x=l).

Figure 8 shows the tip position deflections obtained in the azimuthal movement, provided by: (a) the finite element program (FEM simulation plot in the figure); (b) Estimator (19) (distributed mass model plot in the figure); and (c) Estimator (20) (lumped mass model plot in the figure).

**Figure 9:**
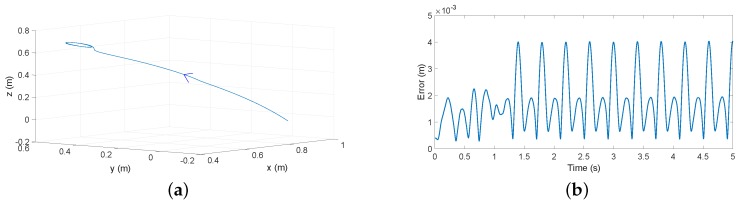
(**a**) 3D trajectory of the tip, calculated by the finite elements software in a movement with only motor control; (**b**) absolute errors of the estimators (19).

Figure 9a shows the tip position when a combined azimuthal-attitude 3D trajectory is carried out with only motor control (a residual undamped vibration can be observed at the end of the trajectory). In this test, the antenna tip is moved $45^{\circ }$ simultaneously in azimuthal and attitude directions in 1.2 s. Figure 9b shows the absolute error $E\left(t\right)=\sqrt{{\left(x^{e}\left(t\right)-x\left(t\right)\right)}^{2}+{\left(y^{e}\left(t\right)-y\left(t\right)\right)}^{2}+{\left(z^{e}\left(t\right)-z\left(t\right)\right)}^{2}}$, $x^{e},y^{e},z^{e}$ being the Cartesian coordinates of the tip given by Estimator (19) and x,y,z the tip position given by the finite element software.

Table 4 shows the differences between the deflections yielded by our estimators and the finite element software in the cases of: (a) the previous azimuthal movement; (b) an attitude movement of $45^{\circ }$ in 1.2 s; and (c) the previous combined movement in 1.2 s. In this table, $E_{m}$ is the average difference between the estimator and the finite element software, and $E_{max}$ is the maximum value of this difference. The deflection differences in an intermediate point of the beam are also shown, in which the deflections were estimated using Equation (14). Moreover, we indicate that, in the previous azimuthal movement, the differences between the tip positions provided by the lumped mass Estimator (20) and the finite element software are $E_{m}=2.3$ mm and $E_{max}=6$ mm.

### 5.2. Experiments on Contact Instant Detection

**Figure 10:**
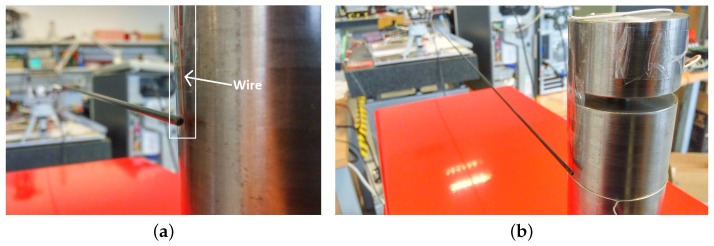
(**a**) Cylinder used in the contact instant detection and the detail of the conductor used to determine this instant; (**b**) object used in the surface exploration experiment.

In this subsection, experiments are carried out in which the antenna performs an azimuthal movement and hits a vertical steel cylinder. In order to exactly know the contact instant, the following device has been assembled. A copper wire has been set vertically, parallel to the cylinder, and very close to its surface, but without touching it, as is shown in Figure 10a. The cylinder is connected to a digital input of a PCI 6229 multifunction data acquisition card of National Instruments, and it is set to zero volts. The conductor wire is set to five volts. Then, at the instant at which the antenna hits the cylinder, it sweeps along the wire towards the cylinder surface closing an electrical circuit. Consequently, a voltage change is produced in the digital input connected to the cylinder, and the exact instant at which the antenna contacts the cylinder is detected.

**Figure 11:**
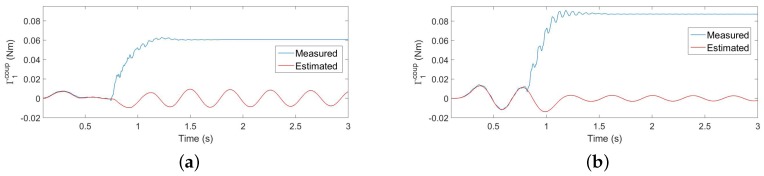
Measured and estimated coupling torques: (**a**) complete control; (**b**) only inner loop control.

In this subsection, all of the plots of the figures are hereafter signals filtered by F(s). Figure 11a,b shows the torque signals provided by the (F-T) sensor ${\vec{\Gamma }}_{s^{\prime }}\left(t\right)$ and the estimates ${\vec{\Psi }}_{s^{\prime }}\left(t\right)+{\vec{\Gamma }}_{s^{\prime }}^{g}\left(t\right)$ provided by the algorithms of Section 4.2 in the collision experiments: one in which the movement is carried out using the complete control system (Figure 11a) and the other using only the motor control (the inner loop; Figure 11b). In this last case, the beam vibrations are not damped during the free movement because the outer loop has not been closed.

**Figure 12:**
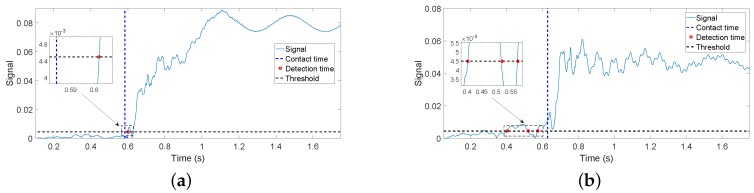
Residues $r_{m}^{f}\left(t\right)$ in the cases of: (**a**) complete control; (**b**) only inner loop.

Figure 12a,b shows the absolute values residues obtained in the same two experiments. The exact instant at which the contact is produced (given by the instant at which the above-mentioned wire closes the electrical circuit) is shown in both figures as vertical discontinuous lines, and the estimation provided by the condition $r_{m}^{f}\left(t\right)>\varepsilon^{\prime }$ proposed in [14] is also marked by a red point. Since the smaller the threshold is the faster the estimation of the contact instant would be, a threshold as small as possible has been chosen. It is determined by the maximum residual that is obtained during a free movement using the complete controller, plus a security margin of 50%. Then, we made $\varepsilon^{\prime }=0.0045$. Figure 12a shows that the delay of the algorithm in estimating the contact instant is 18 ms. However, Figure 12b shows that, since the amplitudes of the residues are bigger in the case of only motor control, the algorithm gives some false detections before the contact is produced (the red points).

**Figure 13:**
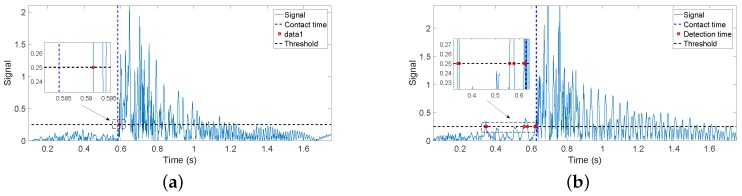
$\left|\frac{dr_{m}^{f}\left(t\right)}{dt}\right|$ in the cases of: (**a**) complete control; (**b**) only inner loop.

Figure 13a,b shows the value of $\left|\frac{dr_{m}^{f}\left(t\right)}{dt}\right|$ obtained in the same two experiments. The exact instant at which the contact is produced is also given in both figures by a discontinuous vertical line, and the estimation provided by Condition (17) is also marked by a red point. Again, a threshold as small as possible has been chosen given by the maximum derivative of the residual obtained during a free movement using the complete controller, plus a security margin of 50%. Then, we made ε=0.25. Figure 13a shows that the delay of the algorithm in estimating the contact instant is 7 ms. However, Figure 13b shows that, since the amplitudes of the derivatives of the residues are bigger in the case of only motor control, the algorithm gives some false detections before the contact is produced.

Table 5 shows the delay in estimating the contact instant with the object of Figure 10 as a function of the relative orientation between the antenna movement and the object surface. The antenna moved in the horizontal plane. The angle formed by the symmetry axis of the cylinder and the horizontal plane is the orientation given in degrees in the table. Ten experiments were carried out for each object surface orientation ($90^{\circ }$ means that the antenna hits perpendicularly to the surface). The average $\bar{t}$ of the obtained delays and the standard deviation σ are shown for each set of experiments. Moreover, the delays in the time estimation are provided for the cases in which the contact instant is obtained from: (a) $r_{m}^{f}\left(t\right)$, which results from filtering the residue (16) with F(s); and (b) $\left|\frac{dr_{m}^{f}\left(t\right)}{dt}\right|$, which results from the time derivative of the filtered residue.

The figures of Table 5 show that:

1. The delay in the estimation using $r_{m}^{f}\left(t\right)$ varies between 21 ms and 46 ms, depending on the relative orientation, while the delay in the estimation using $\left|\frac{dr_{m}^{f}\left(t\right)}{dt}\right|$ varies between 5 ms and 6.5 ms. Then, it can be concluded that the second estimator is significantly faster than the first one.
2. The influence of the relative orientation on the estimation delay is significantly higher in the first estimator than in the second.
3. In the case of using $r_{m}^{f}\left(t\right)$, the delay in the estimation of the contact instant grows as the relative orientation decreases because the component of the force exerted by the antenna that is normal to the object surface diminishes as the relative orientation decreases. Then, the slopes of the filtered residues shown in Figure 12 diminish, and more time is required to reach the detection threshold. Experiments have shown that this phenomenon does not happen in the case of using the derivative of the residue to detect the contact instant.

### 5.3. Experiments on Contact Point Detection

An experiment was carried out in which the point of contact of the antenna with the object of Figure 10 was estimated using Algorithm (18). The results obtained are shown in Table 6. Experiments were performed for different relative orientations between the antenna movement and the object surface. The antenna moved in the horizontal plane. The angle formed by the symmetry axis of the cylinder and the horizontal plane is the orientation given in degrees in the left column of the table. The upper row of the table indicates the position of contact along the sensor rod as measured from the center of rotation. Two sets of data are provided in this table: (a) contact position estimates from simulations using our finite element program; and (b) contact position estimates obtained in the experiments. From this table, it can be observed that:

1. The error in the estimation of the contact position increases as the relative orientation between the cylinder symmetry axis and the direction of the antenna movement diminishes. This trend is shown both in the simulated and the experimental results.
2. The error in the estimation of the contact position decreases as the contact point is nearer to the center of rotation of the antenna. This trend can be also noticed in both the simulated and the experimental results.
3. For relative orientations of $60^{\circ }$ or more, the average error in the estimation of the contact position is about 2 cm.

Other experiments were carried out in which a profile was recognized. Figure 10b shows the object to be explored. It is a vertical cylinder with a horizontal slot. One of its vertical sides is looked over by the antenna in the experiment, in which the antenna hits horizontally the object at heights equally spaced. Two experiments were carried out: one in which the height equally spaced is 20 mm and another in which this height is 10 mm. For each experiment, two cases are performed: one in which the complete control system is used and another in which only the motor control is used. The effectiveness of the control scheme and the estimation algorithm was verified using the optotrack camera-based optical tracking system as an external sensor. The reference frame of this external sensor was located at the base of the tip and in the direction of the fixed frame XYZ of Figure 1. The initial condition of every maneuver was chosen as $\phi_{1}=0$ and $\phi_{2}=0$. The ground of the object, the position of the slot [57–67 mm] and its thickness (10 mm) are measured before doing the experiments.

**Figure 14:**
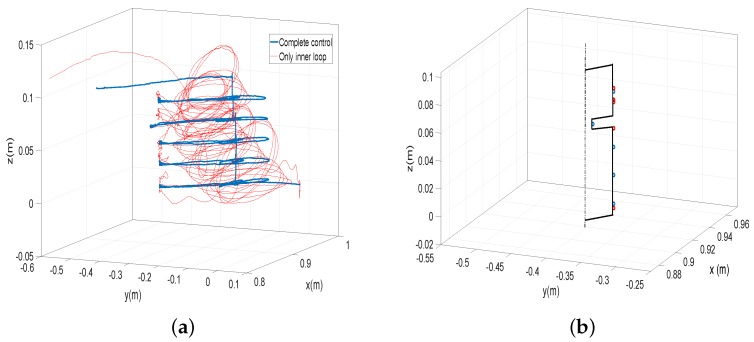
(**a**) Trajectories performed by the tip of the antenna in the profile recognition experiment; (**b**) detail of the points estimated by the antenna (in blue with complete control and in red with only inner loop control).

Figure 14a shows the tip trajectories in the first experiment (height spaced 20 mm), measured with the camera-based optical tracking system. In this figure, the trajectory performed using the complete control is shown with blue color, and the trajectory using only the motor control is shown with red color. In Figure 14b, the contacted points are marked over the real object profile (in perspective), blue or red circles in the cases of using the complete control or only the motor control, respectively. Table 7 shows the *z* coordinates measured with the external sensor and the $z^{e}$ coordinates of the contacted points determined by the Equations (19) from measures of the motor angles and the coupling torques (then, according to Table 4, they are determined with an average error of about 0.5 mm because the movements of the experiments are mainly azimuthal and attitude movements).

**Figure 15:**
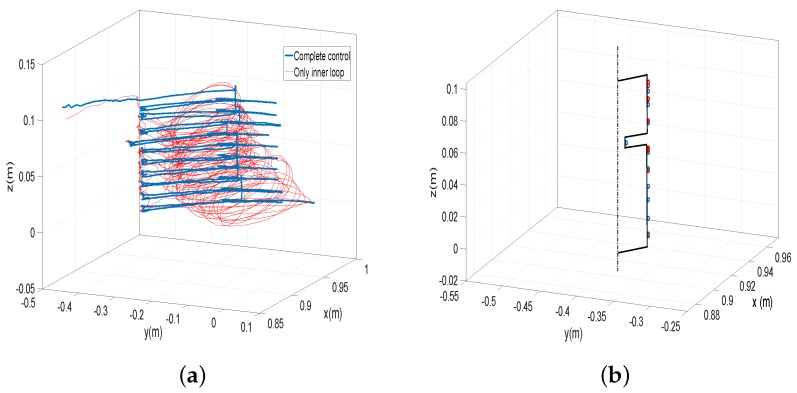
(**a**) Trajectories performed by the tip of the antenna in the profile recognition experiment; (**b**) detail of the points estimated by the antenna (in blue with complete control and in red with only inner loop control).

Figure 15a shows the tip trajectories in the second experiment (height spaced 10 mm), measured with the camera-based optical tracking system, and Figure 15b the contacted points measured also with this device. Table 8 shows the *z* coordinates measured with the external sensor and the $z^{e}$ coordinates of the contacted points determined by the Estimators (19).

## 6. Discussion

### 6.1. Vibration Control System

Several control systems that effectively damp vibrations in flexible link robots have already been proposed. Some of them close a feedback loop using the measurements of strain gauges placed close to the robot joints. Among the references cited in this paper that feedback this signal, we mention that: [13] implements PID controllers with low pass filters; [14] implements GPI controllers; [23] implements PI controllers; and [15] *P* controllers. All of these controllers have zero relative degree, which means that high frequency unmodeled dynamics and noises are not damped. Our controllers effectively damp the lower vibration modes, and moreover, since their relative degree is α, the magnitude of their frequency responses tends to zero at high frequencies, thus highly reducing the effects of high frequency unmodeled dynamics and noises.

Since high frequency unmodeled modes and noises (that appear in the strain gauge sensor signal) influence much more the dynamics of our slender antenna than the dynamics of heavier and more rigid flexible link robots, the controller described in this paper is especially appropriate for this application.

### 6.2. Beam Position Estimation

Simulated tests have shown that the proposed estimators (19) are a good compromise between accuracy and complexity. They are very simple and provide estimations of the tip position that are quite accurate: in relatively fast movements, the average error at the tip is around 2 mm, and the maximum error is 4 mm. These errors in our 1 m-long very flexible antenna are considered very small. Another estimator with a mathematical structure similar to ours is Equation (20). This one yielded about five-times higher errors than Equation (19) in the tip position estimation. Note that the coefficient that multiplies the coupling torque makes the main difference between these two algorithms: in one case, it is obtained from assuming a distributed mass beam and truncating to the first vibration mode, and in the other case, it is obtained from assuming a massless beam with a tip lumped mass, which yields a single vibration mode (without any need of truncating a model). These simulations show that a lumped mass model is inappropriate to describe the beam part of our antenna dynamics. These tests show that the estimations of the deflections of intermediate points of the beam provided by our Algorithm (14) are more accurate than the ones obtained at the tip. This proves that Equation (14) describes adequately the bending shape of the antenna during its motion.

Besides this, the dynamic model experimentally obtained of the beam shows three vibration modes, i.e., a linear dynamics of sixth order (see Figure 4 and Expression (2)). This suggests that, in order to accurately estimate the tip deflections, two observers of order six (or five if reduced order observers were used) should be implemented, one for each degree of freedom. In this sense, our estimators are much more simple than the observers of the beam dynamics and yield enough accuracy for many applications.

### 6.3. Contact Instant Estimation

The instant and location of contact/impact in mechanical structures can be very quickly estimated by detecting the acoustic waves that propagate through the 3D structure, e.g., [26]. In particular, a patent on detecting the instant and the point of impact on a flexible link made of aluminum by sensing acoustic waves using strategically-located piezoelectric sensors was developed in [27]. The velocity of the acoustic wave is direction-dependent in anisotropic materials. Even for isotropic materials, the wave speed may be also slightly influenced by structural thickness, stiffening beams or other parameters. In the case of carbon fiber, the acoustic wave velocity is always higher than 2000 m/s, e.g., [28], which means that the impact can be detected with a delay lower than 0.5 ms in our link 0.98 m long. However, piezo-actuators need a flat surface in which they have to be stuck. We are using a very thin rod with a circular cross-section of radius of 1 mm in our sensing antenna (all of the sensing antennae are made with very thin rods). Then, the curvature of the antenna is very large, and it is not possible to fix any piezo-actuator on its surface. Moreover, these sensing antennae are exposed to large deflections when they touch an object. This produces adverse effects on the piezoelectric functioning, and even this may cause the piezoelectric being detached from the rod.

We use therefore (F-T) sensors instead of piezoelectric sensors, which are less sensitive and have a lower frequency bandwidth than these, but can be integrated in the system, as is shown in Figure 1. Subsequently, a comparison is carried out among methods that estimate the instant of impact on mobile structures by using sensors that measure elastic deformations. We consider some impact detection systems used in multi-link robots, though these are different from our single link device. This is because though our antenna and the multi-link robots have different mechanical configurations, they share the physical principle that is used by their sensors to estimate the contact instant.

Estimation of the contact instant in rigid link robots (these may have some elasticity in the joints) was carried out in [12] by detecting if a threshold of a residual of a generalized momentum is surpassed. This method needs to compute the generalized momentum of the robot and uses the torques submitted by the actuators to the robot links, which are directly measured by load side joint torque sensors. This method has the drawback that it needs the numerical differentiation of the joint angles, which may introduce noise and inaccuracies.

Estimation of the contact instant in flexible link robots was proposed first in [13]. A mechanism was developed that detected contact when tip position and velocity errors simultaneously surpassed some thresholds. Tip deflection was estimated from the torques submitted to the links, which were directly obtained from strain gauges placed in the robot structure. This method has also the previously-mentioned problem of having to differentiate some measurements. In [14], a method was proposed that did not need such numerical differentiations because the contact instant was detected when the difference between the measured coupling torque and the coupling torque predicted by a model surpassed a threshold. Finally, a method was proposed by [15] that detected the contact instant by implementing a method similar to the one proposed in [12], but using strain gauge measurements instead of load side joint torque sensors to obtain the torques delivered by the actuators to the robot links.

Compared to the previous contact detection mechanisms, the proposed Algorithm (17) presents several advantages when applied to our sensing antenna device:

1. Experiments reported in [13] showed that the delay in the contact estimation of that method was about 300 ms, much larger than the 7 ms provided by the proposed method.
2. The experiments carried out here with the estimator that is based on the magnitude of the residue of the coupling torque ($r_{m}^{f}\left(t\right)>\varepsilon^{\prime }$), proposed in [14], yielded delays in the contact instant estimation more than three-times larger than with the estimator proposed here: about 20 ms.

The instant of contact has also been estimated in other artificial antennae that mimic insect behavior [29]. In these experiments, a two-axis acceleration sensor placed at the tip of the antenna was used, which measured the link vibrations. The information about contact was obtained from processing the vibration frequencies. However, the delay in the estimation of the instant of contact was not given.

The delay in detecting the contact depends on the estimation algorithm, but also mainly on the slope of the growth of the coupling torque once the hit has been produced. The steeper this growth is, the faster the contact estimation would be. This slope is defined by the rigidity of the impacted beam. Then, the contact can be detected more quickly in beams with high rigidity than with low rigidity. The beam of our antenna is very elastic (it is made of carbon fiber with a very small cross-section), in order to facilitate the object recognition, while robots with flexible links have significantly higher rigidity (many of them are made of aluminum, which has a Young modulus value about half of our carbon fiber, but they have cross-sections of much higher inertia moment values). This means that we would expect a significantly larger delay in estimating the contact instant in our antenna experiments than in experiments with flexible link robots (e.g., [13,14,15]) if we used the residue $r_{m}^{f}\left(t\right)$. However, since we use the time derivative of this signal, a change in the slope of $r_{m}^{f}\left(t\right)$ from nearly zero in the free movement to a large value in the constrained movement caused by the growth of the contact torque would produce a very quick surpassing of the contact detection threshold. Then, our algorithm yields a very small delay in the estimation of the contact instant, which is not much affected by the rigidity of the beam. This delay is smaller than the delays obtained in flexible robots [13,14].

**Figure 16:**
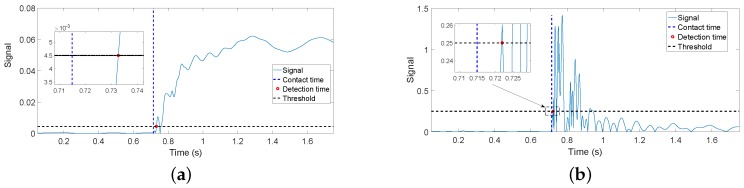
Contact instant detection obtained from a simulation with a finite element program of the controlled antenna: (**a**) using $r_{m}^{f}\left(t\right)$; (**b**) using $\left|\frac{dr_{m}^{f}\left(t\right)}{dt}\right|$.

Regarding the physical feasibility limit in the detection of an impact in a flexible link and the closeness of the performance of our algorithm to this limit, it is mentioned that:

1. Impact detection can be achieved detecting the acoustic wave that propagates through the structure using piezoelectric sensors in a time far below 1 ms, as cited before.
2. The dynamics that can be detected by an (F-T) sensor, which has significantly lower bandwidth than piezoelectrics, was simulated using our finite element program, in which the link was modeled by 100 flexible elements. Numerical simulations of the contact of the completely controlled antenna with a rigid object were carried out. The same thresholds as in Section 5.2 were used. These simulations yielded the responses drawn in Figure 16. Figure 16a shows the simulation of residue $r_{m}^{f}\left(t\right)$ and Figure 16b the simulation of $\left|\frac{dr_{m}^{f}\left(t\right)}{dt}\right|$. It can be observed that these plots are very similar to the results of the experiments shown in Figure 12a and Figure 13a, respectively. The delays obtained in the different cases are presented in Table 9, which shows that experimental and simulated results are in good agreement. Moreover, these simulated results show that the delay attained in estimating the contact instant is close to the physical limit and that there is little margin to further reduce this delay. For example, diminishing the threshold in Figure 16b would highly increase the risk of false detections while the attained reduction in the detection delay would be very small owing to the very sharp growing shape of the presented response.

Regarding the computation features of the proposed estimator, we remark about the following issues:

1. Since the movement of our slender and lightweight link produces very small torques, these can be neglected if compared with the reaction forces-torques produced in a contact situation. Then, a very significant reduction in the real-time computation burden can be achieved by using the magnitude of the filtered torque provided by the (F-T) sensor minus the filtered torque produced by the gravity, $s_{m}^{f}\left(t\right)$ (we define $s\left(t\right)={\vec{\Gamma }}_{s^{\prime }}\left(t\right)-{\vec{\Gamma }}_{s^{\prime }}^{g}\left(t\right)$; $s^{f}\left(t\right)$ is the result of passing the components of the vector s(t), separately, through the linear filter F(s), and $s_{m}^{f}\left(t\right)$ is the magnitude of the vector $s^{f}\left(t\right)$), instead of the residue $r_{m}^{f}\left(t\right)$, or its time derivative $\left|\frac{ds_{m}^{f}\left(t\right)}{dt}\right|$ instead of $\left|\frac{dr_{m}^{f}\left(t\right)}{dt}\right|$. In these cases, a dynamical model does not need to be computed in real time, and the required computations are very simple, basically being the calculation of the magnitude of a filtered vector and, if Estimator (17) were used, a numerical differentiation.
2. Table 9 shows the delays achieved in the contact instant estimation by using the previous two simplified estimators. The threshold used in the impact detection with $\left|\frac{ds_{m}^{f}\left(t\right)}{dt}\right|$ is equal to the one used with $\left|\frac{dr_{m}^{f}\left(t\right)}{dt}\right|$. It can be noticed that the estimation delay is approximately the same in these two estimators when applied to both experimental and finite element simulation data. However, the delay attained using the estimator based on $s_{m}^{f}\left(t\right)$ is larger than the one attained with $r_{m}^{f}\left(t\right)$. This is because the torques measured by the (F-T) sensor during an antenna free movement have higher amplitudes than their corresponding residues, which forces us to use a higher threshold in order to avoid false detections, and once the contact has been produced, the reaction torque requires more time to reach this new threshold.

**Figure 17:**
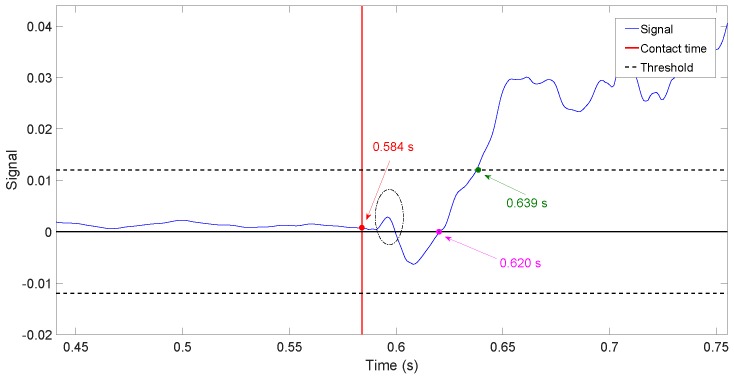
Experimental values of the vertical component of the measured torque in a horizontal movement with impact.

The transfer function between the coupling torque and the tip angle of the antenna was obtained analytically including three vibration modes. The zeros of this transfer function are ±49, ±216.2,±j100.4. Then, this transfer function is non-minimum phase, and it also would be the transfer function between the contact force at the tip and the torque at the base of the antenna. Figure 17 shows the experimental values of the vertical component of the measured torque in a horizontal movement with impact. The threshold applied in the experiments that used the torque measurements, and that were reported in the fourth item of the previous paragraph, is also drawn. This figure shows the non-minimum phase behavior, i.e., immediately after the contact has happened, a slight undershot in the (F-T) sensor measurement appears, and subsequently, the response moves towards the expected value.

Sometimes, the previous non-minimum phase behavior is approximately modeled as a delay. The zero in 49 (see Equation (2)) is dominant with respect to the other zeros located in the right half-plane (it is more than four-times closer to the imaginary axis than the other zeros). This zero gives an idea of the time needed by the deflection to propagate from one end to the other of the link, e.g., [30]. If a delay were approximated by a first-order Padé approximation $e^{-Ts}\approx \left(1-Ts/2\right)/\left(1+Ts/2\right)$, this time delay would be T/2=1/49→T=41 ms. If we assume that this delay is approximately the time needed by the response to finish its undershot phase and start growing in the appropriate direction, i.e., the time required by the torque to cross the zero axis and start continuously growing, this time is given by the magenta point marked in Figure 17. This figure shows that the time elapsed from the contact instant to that zero crossing instant is 36 ms, which is similar to the delay previously obtained from the dominant zero. The instant at which the contact is detected is also shown, marked by a green point. In this figure, the small positive peak that appears at the very beginning of the contact and that is surrounded by an ellipse is the part of the response used by the estimator $\left|\frac{ds_{m}^{f}\left(t\right)}{dt}\right|>\varepsilon$ to detect the contact instant. It shows that the contact instant detection is significantly sped up if the time derivative estimators are used.

### 6.4. Contact Point Estimation

The performance of the standard control system used for sensing antennae, which is based on only motor control, has been compared with our complete control system that removes antenna vibrations. Figure 14a and Figure 15a show that the apparently “chaotic” vibrations that appear using a standard motor controller (red trajectory), as a consequence of the vibrations existing during the free motion, which are highly excited by the impacts of the antenna with the object, are quickly removed in the case of using the complete control system yielding smooth and well-controlled trajectories (blue trajectory).

The task programmed for the antenna was to hit the object horizontally performing regular jumps of 20 mm and 10 mm in the vertical coordinate before each approaching maneuver to the object. From Table 7 and Table 8, it is obtained that the mean of the differences between consecutive contacted points (in the *z* coordinate) is 20.1 and 10 mm in the case of using the complete control and 21.2 mm and 9.2 mm in the case of using only the motor control. Moreover, the standard deviation of these differences with respect to 20 mm and 10 mm is 0.8 mm and 1.2 mm in the first case and 22.9 and 13.2 mm in the second. These figures show that regularity in the points contacted is much better achieved by using the complete control system that damps the vibrations than by using only the motor control. This issue has a relevant impact in the performed experiment: Figure 14b and Figure 15b show that the slot is detected in the two experiments in the case of using the complete control system, while in the case of using only motor control, the slot is not detected in any of them. These two experiments were carried out ten times each. It was obtained that, using the control system, the slot was detected in all the trials. However, using only motor control, the slot was not detected in any trial due to the vibration of the antenna.

Table 7 and Table 8 also show that, in the case of using the complete control, the $z_{e}$ coordinates of the contacted points determined by the estimators (19) are very similar to the measurements provided by the camera-based optical tracking system. It is obtained that the means of the absolute errors between the measures of the contact points provided by the camera-based optical tracking system and the estimators (19) are 0.5 mm and 1 mm in the cases of regular jumps of 20 mm and 10 mm, respectively, in the vertical coordinate. The standard deviation of these errors is 0.5 mm in both experiments. However, these errors are much bigger in the case of using only motor control. In this last case, the mean of the absolute errors between the measures of the contact points provided by the external cameras and the estimators are 7.5 mm and 5.5 mm in the cases of regular jumps of 20 mm and 10 mm, respectively, in the vertical coordinate. The standard deviations of these errors are 5.5 mm and 4 mm, respectively.

Errors obtained using the complete control system are therefore admissible, if the accuracy of the estimators (19) are taken into account, whereas errors obtained in the case of using only inner loop control are not admissible, according to Table 4. This feature can be explained by the fact that, using only inner loop control, the movement of the tip of the antenna shows apparently chaotic vibrations that produce big deflections. These deflections may exceed the 10% limit usually allowed for the beam deflections to be considered geometrically linear (i.e., the case in which the tangent and the sine can be approximated by the angle). Our estimators may exhibit significant errors under very large deflections that violate the geometrically-linear deflection condition. In our experiments, in the case of using the complete control, the deflections in the contact instants are around 4 cm (4.1% of the total length of the beam). This deflection is due mainly to the static deflection prompted for the gravity and is far away from the before limit. However, in the case of using only inner loop control, the deflections in the contact instant have a range between 7 cm and 10 cm (between 7.2% and 10.2%), which is a range in which linear models begin to show small inaccuracies.

## 7. Conclusions

We conclude from the previous discussion that the estimation algorithms developed in this article are computationally efficient enough to be implemented in our sensing antenna device, which uses a computer of limited capability, in real-time tasks, while achieving a reasonable accuracy. Moreover, we conclude that using a control system that, besides moving the motors, cancels the antenna vibrations, significantly improves the performance of the before algorithms and the overall object detection and recognition tasks.

One foreseen application is obstacle detection in mobile robot navigation. The link length is 0.98 m. Since the height of the robot platform where the antenna is placed is about 30 cm, simple trigonometry allows us to state that our system is able to detect an obstacle on the ground placed 0.9 m beyond the vehicle in the direction of the movement. Since our mobile robot moves at a speed of about 0.4 m/s, this system detects the obstacle at a distance sufficient to permit the change of trajectory or the complete stop of the vehicle before hitting the obstacle. Moreover, some simple recognition tasks of the obstacle can be carried out by our system, which is able to estimate points on the surface of the obstacle with a precision of 2 mm.

Finally, it is highlighted that these are our first results on object detection with a controlled antenna. The closed-loop control system removes the vibrations satisfactorily, and the contact instant detector is enough accurate. However, it is mentioned that several issues of the estimation mechanisms can be improved and will be the object of our future research:

1. The 3D position of the tip of the link is estimated with an average accuracy of nearly 2 mm in combined azimuthal-zenithmovements (intermediate points are estimated with higher accuracies). The developed estimator is simple and only uses present sensor measurements. A dynamic observer can improve these estimations by using also past measurements in combination with a dynamic model of the link.
2. Algorithm (18) that estimates the point of the antenna at which the contact is produced has an accuracy of about 2 cm (2% of the link length). This is accurate enough for locating an obstacle in the trajectory of a mobile robot, but it is insufficient for recognizing an object. Moreover, it only yields accurate estimates if sliding on the object surface does not happen and friction force does not appear. Then, another dynamic observer is required that, based on past measurements, increases the accuracy of the contact point location algorithm and estimates if sliding is produced, correcting the effect of surface tangential forces on the estimation of the contact point.
3. It has been shown that contact in which the relative orientation between the direction of the antenna movement and the vector normal to the object surface is higher than $45^{\circ }$ yields inaccurate estimates. Then, an antenna trajectory planner is needed that, as a function of the estimates of previous contacted points, defines the future trajectory of the antenna in order to hit the object in an appropriate direction.
4. An object recognition system has to be developed. It implies the regularization of the obtained data, object estimation and pose estimation, based on an object database. A system similar to the one proposed in [31] for computer vision could be developed.

## Figures and Tables

**Table 1:** Beam characteristics.

Parameters Flexible Link	Values	Unit
Length *l*	0.98	m
radius *r*	1	mm
Cross-section $C_{S}$	3.14	mm^2^
Section inertia moment *I*	0.785	mm^4^
Young modulus *E*	$115\times 10^{9}$	$\frac{N}{m^{2}}$
Linear density ρ	4.7	g/m
Link mass *m*	4.6	g
**First Vibration Mode**		
Natural frequency ω	16.6	s^−1^
Damping coefficient $\epsilon_{1}$	0.009	s^−1^

**Table 2:** Parameters of the motors.

Parameters	$\hat{K_{i}}$	${\hat{J}}_{i}$	${\hat{\nu }}_{i}$	$\vartheta_{i}^{\mathrm{nlc}}$	$\vartheta_{s,i}$	$n_{i}$
Motor	$\frac{\mathrm{Nm}}{V}$	$\mathrm{kg}\cdot m^{2}$	Nm·s	V	V	
$\theta_{1}$	0.003	$6.18\times 10^{-7}$	$3.04\times 10^{-6}$	0.48	1.2	100
$\theta_{2}$	0.003	$1.85\times 10^{-7}$	$2.85\times 10^{-6}$	0.42	1.2	100

**Table 3:** Control parameters.

DOF	Poles	$a_{0,i}$	$a_{1,i}$	$a_{2,i}$	$b_{0,i}$	$b_{1,i}$	$h_{i}$
*i*	$p_{i}$						
$\theta_{1}$	−60	26,7000	8900	74.2	8900	347	235.1
$\theta_{2}$	−60	79,920	2664	22.2	2664	89.7	224.6

**Table 4:** Errors of the estimates given by Equation (19).

Errors	Azimuthal	Azimuthal	Attitude	Attitude	Combined	Combined
	(*x* = l)	(*x* = l/2)	(*x* = l)	(*x* = l/2)	(*x* = l)	(*x* = l/2)
$E_{m}$ (mm)	0.33	0.16	0.63	0.24	1.8	0.55
$E_{max}$ (mm)	1.3	0.47	1.2	0.62	4	1.2

**Table 5:** Delay in the detection of the time of contact.

	$\bar{t}\;\left(90^{\circ }\right)$	$\sigma \;(90^{\circ })$	$\bar{t}\;\left(75^{\circ }\right)$	$\sigma \;(75^{\circ })$	$\bar{t}\;\left(60^{\circ }\right)$	$\sigma \;(60^{\circ })$	$\bar{t}\;\left(45^{\circ }\right)$	$\sigma \;(45^{\circ })$
$r_{m}^{f}\left(t\right)$	21.2 ms	3.5 ms	41.4 ms	1.5 ms	42 ms	3.4 ms	46.2 ms	2.2 ms
$\left|\frac{dr_{m}^{f}\left(t\right)}{dt}\right|$	6.6 ms	1.9 ms	4.8 ms	1.9 ms	6.4 ms	1.9 ms	6.5 ms	3.8 ms

**Table 6:** Identification of the contact point of the antenna for different relative orientations (positions are given in cm).

	FEM Software			Experimental		
	95	74	56	95	74	56
$90^{\circ }$	94.9	73.7	55.8	96	71	55.5
$75^{\circ }$	94	73.5	55.7	91	71	55.5
$60^{\circ }$	93.9	73.9	55.9	97	73	57
$45^{\circ }$	93.7	74.7	56.4	85	77	58

**Table 7:** Vertical coordinates of the estimates given by Equation (18) of the points contacted by the antenna on the object’s surface.

Point	1	2	3	4	5
*z* with complete control (mm)	0.1	20.8	40.8	59.7	80.5
*z* with only motor control (mm)	−3.0	55.7	74.7	73.2	81.6
$z^{e}$ with complete control (mm)	0.0	21.5	42.5	60.0	80.0
$z^{e}$ with only motor control (mm)	−18.0	67.5	75.5	76.0	73.5

**Table 8:** Vertical coordinates of the estimates given by Equation (18) of the points contacted by the antenna on the object’s surface.

Point	1	2	3	4	5	6	7	8	9	10
*z* with complete control (mm)	0.1	9.8	21.5	30.1	41.1	51.2	59.6	69.8	81.3	89.6
*z* with only motor control (mm)	−1.1	40.0	52.6	53.1	54.2	71.0	84.5	84.7	80.5	81.3
$z^{e}$ with complete control (mm)	0.0	10.5	21.0	28.5	39.5	52.0	59.0	70.0	80.0	88.5
$z^{e}$ with only motor control (mm)	1.0	41.0	54.0	51.0	42.5	62.0	79.0	74.5	84.0	91.0

**Table 9:** Delay in the estimation of the instant of contact obtained in simulated and experimental data.

	Simulated	Experimental
using $r_{m}^{f}\left(t\right)$	Figure 16a: 17 ms	Figure 12a: 18 ms
using $s_{m}^{f}\left(t\right)$	53 ms	55 ms
using $\left|\frac{dr_{m}^{f}\left(t\right)}{dt}\right|$	Figure 16b: 7 ms	Figure 13a: 7 ms
using $\left|\frac{ds_{m}^{f}\left(t\right)}{dt}\right|$	7 ms	7 ms
